# Spatial and simultaneous representative seroprevalence of anti-*Toxoplasma gondii* antibodies in owners and their domiciled dogs in a major city of southern Brazil

**DOI:** 10.1371/journal.pone.0180906

**Published:** 2017-07-21

**Authors:** Aline do Nascimento Benitez, Felippe Danyel Cardoso Martins, Marcelle Mareze, Nelson Jessé Rodrigues Santos, Fernanda Pinto Ferreira, Camila Marinelli Martins, João Luis Garcia, Regina Mitsuka-Breganó, Roberta Lemos Freire, Alexander Welker Biondo, Italmar Teodorico Navarro

**Affiliations:** 1 Laboratory of Zoonoses and Public Health, Londrina State University, Londrina, Paraná, Brazil; 2 Department of Preventive Veterinary Medicine and Animal Health, University of São Paulo, São Paulo, São Paulo, Brazil; 3 Department of Preventive Veterinary Medicine, Londrina State University, Londrina, Paraná, Brazil; 4 Department of Veterinary Medicine, Federal University of Paraná, Curitiba, Paraná, Brazil; Wageningen Universiteit, NETHERLANDS

## Abstract

Toxoplasmosis, caused by *Toxoplasma gondii*, has traditionally been considered an important water and foodborne protozoonosis with important public health considerations. Although felids play a well-established role as definitive hosts, canine epidemiological involvement in the parasite’s life cycle remains questionable and controversial. The increasing closeness of the human-dog bond, particularly seen in urban settings, has been recognized as a historically unprecedented worldwide movement. Sharing daily lives in the same households, dogs may be exposed to similar associated risks of *T*. *gondii* infection as their owners. Thus, epidemiological assessment of the intra-domiciled environment, especially among socio-economically different human populations, may provide novel information regarding the actual role of dogs in animal and human toxoplasmosis. Despite spatial approaches being recently used for other water and foodborne diseases, no study has been conducted on the simultaneous spatial seroprevalence of both human and animal IgG anti-*T*. *gondii* antibodies in urban areas of major cities. Accordingly, the aim of the present study was to assess the seroprevalence and associated variables of *Toxoplasma* infection in owners and their domiciled dogs in Londrina, southern Brazil. Human and canine seroprevalence rates and variables associated with seroprevalence were investigated through representative random sampling among 564 households, which included 597 owners and 729 dogs. Overall, statistically significant differences between the seroprevalence of human and dog anti-*T*. *gondii* antibodies were found by Immunofluorescence Antibody Testing in 248/597 (41.54%) owners and 119/729 (16.32%) dogs. Through multiple analysis, significant concomitant variables for seropositivity of household individuals (people and dogs) were determined, including public sewer service, yard cleaning frequency, and having a dirty yard. Although no statistically significant multiple logistic model was observed among owners, univariate analysis detected associations with monthly income, soil contact, and occupation. Among dogs, the absence of other dogs and the absence of a dirty yard were concomitant significantly protective associated factors. Age differences between seropositive and seronegative individuals was significant only for human beings, with the median age of negative individuals significantly higher than positive individuals. Although no spatial clusters were identified for humans or residences, a significant cluster was identified for dogs. In conclusion, characteristics of urban toxoplasmosis may include significantly higher owner seroprevalence than their owned dogs, with canine seroprevalence directly associated with having more dogs and a dirty backyard, and spatial differences in both human and dog exposures. Although not a good indicator for human foodborne diseases, dogs may be a reliable sentinel for environmental infection. Moreover, such a holistic approach may provide crucial information for more focused prevention and monitoring programs, particularly in households with multiple pets and trash-filled backyards.

## Introduction

*T*. *gondii* has been described as an obligate intracellular parasite capable of infecting warm-blooded animals. The only known definitive hosts are the Felidae family, which may eliminate environmentally resistant oocysts, and a wide range of intermediate hosts including human beings [1,2]. Environmental settings may play an important role in Toxoplasma transmission and persistence, since oocysts shed within feces still require favorable conditions to become infectious [3].

Human beings may be primarily infected by *T*. *gondii* via food or water intake, mainly through raw or uncooked meat containing cysts, unwashed food, or contaminated water carrying oocysts [2]. Congenital transmission may also occur during pregnancy, which has been observed in women and other female mammals including bitches [2,4]. While consumption of raw or uncooked meat may not influence toxoplasma infection, social vulnerability in pregnant women has reportedly been shown to increase the risk of toxoplasmosis [4,5]

Although frequently asymptomatic, toxoplasmosis may be chronically reactivated by severe immunosuppression, causing toxoplasmic encephalitis [6]. In addition, congenital toxoplasmosis may cause visual, hearing, neuromotor or learning impairment in up to 20% of infected patients, and clinical disease with hydro/microcephalia, chorioretinitis, cerebral calcification and mental retardment in 13% of cases [4]. Neuropsychiatric disorders have been recently reported, ranging from psychoses and neuroses to Alzheimer's, Parkinson's diseases and suicidal behavior [7,8].

Different manifestations of toxoplasmosis have been considered as important threats to public health, particularly due to the significant amount of years of life lost or years lived with disability (YLDs) from physical sequelae of disease [9]. Toxoplasmosis was estimated by the WHO [10] as the second most common foodborne parasitic disease in 2010, with 10.3 million cases (95% UI 7.40–14.9 million worldwide), resulting in significant morbidity and mortality in vulnerable populations, along with helminth foodborne diseases [11].

The human–animal bond, recognized as a special relationship between people and companion animals, has led to the hypothesis that humans and animals living in the same household, sometimes sharing the same bed, may be closely related from an epidemiological perspective [12]. Dogs may be infected and participate in several other zoonotic life cycles, posing direct public health risks [13], and have been epidemiologically linked as a risk factor for *T*. *gondii* infection in human beings [14–17]. However, in contrast to the well-established involvement of cats as definitive hosts that are important for environmental contamination but not directly as companion pets [18], the epidemiological importance of dogs in urban toxoplasmosis remains controversial [19,20].

Dogs have been imputed with different functions in the toxoplasmosis cycle with varying roles, including host, the final link of the infection chain, carrier (14) that are contagious but asymptomatic [21], or disease sentinels [19,22]. In addition, a dog’s exposure to soil, house dust and airborne particles was indubitably higher when compared to the exposure of their owners [23]. Thus, simultaneous analyses of owners and their dogs may provide a unique opportunity to assess the exposure and epidemiological involvement of certain zoonoses in intrahousehold environments.

The growing One Health movement has been addressing the ecosystem in a coordinated manner, interrelating human, animal, and environmental health [24,25]. In such a scenario, an inter-disciplinary approach may be required for better understanding of the spreading, fluctuations, epidemics and outbreaks of infectious pathogens, particularly zoonoses [26]. *T*. *gondii* may provide a practical example of complex pathogen transmission involving several hosts and environments and may illustrate the importance of a One Health approach to disease ecology and epidemiology [3].

In addition to a holistic approach, recent studies have also shown geoprocessing to be an important tool for better interpretation of spatial characteristics and dynamics of infectious diseases [27,28]. In a northern state ranked fourth on the total Brazilian beef export, seropositive clusters for toxoplasmosis in beef cattle have shown a low impact of the disease despite a widespread undercooked barbecue tradition [29]. In other situations, the use of a spatial approach as an epidemiological tool has been strongly suggested to improve associated risk analysis and prevention of *T*. *gondii* through identifying landscape characteristics and mapping environmental contamination [28,30–32].

Although One Health and geoprocessing have reportedly facilitated the increased comprehension of several zoonotic diseases [33–36], no such approach has been performed to date on the spatial prevalence of toxoplasmosis in dogs and their owners, particularly in major urban cities. Accordingly, the aim of the present study was to simultaneously assess the seroprevalence and associated variables of *Toxoplasma* infection in owners and their domiciled dogs in Londrina, a city with half a million people in southern Brazil. In addition, seroprevalence results and variables associated with infection in owners and dogs were mapped and statistically analyzed.

## Materials and methods

This study has been approved by the National Human Ethics Research Committee (protocol number 1,025,861/2014) and the Animal Use Ethics Committee (protocol n° 181/2014), both through the State University of Londrina, southern Brazil. In addition, the present study has also been approved by the Londrina City Secretary of Health and officially included as part of the annual activities.

Londrina (23°18′36″S and 51°09′46″W) has been the county seat of a metropolitan area and the second biggest city of Parana State, southern Brazil. This city was selected due to its high urban area of 97.00%, high urban population of 543,003 habitants (ranked 18^th^), and high human development index (HDI) of 0.841 (ranked 145^th^ out of 5,570 total Brazilian cities) [37]. Londrina urban area was concentrated at the time of survey, with a total of 161,144/164,898 (97.72%) municipality households [38].

No data on seroprevalence of IgG anti-*T*. *gondii* antibodies were available at the time of the survey, either for human or dog populations. Thus, calculations for size sampling were designed with an expected 50% prevalence, 5% accuracy, 95% confidence level, and an initial population of 161,144 households, for a final minimum sampling size of 384 individuals, with visits distributed only in urban households using a freely available software (EpiInfo 3.5.2, CDC, Atlanta, GA, USA) [39].

A sampling of 461 households was ultimately calculated due to an additional 20% (77) safety margin to account for potential refusal to participate, dog aggressiveness, sample clotting or hemolysis, closed households and commercial properties. Multi-professional field teams including nurses and veterinarians were formed and randomly performed house-to-house visits, following designs by conglomerate. The sorting of households was performed using commercial software (BioEstat 3.0, Belém, PA, BRA) [40], using four households per block for a calculated total of 115 (461/4) blocks, with two blocks per city section of urban planning (115/2), for a total of 58 city sections covered.

Researcher groups were coordinated and guided by professionals from the City Secretary of Health, which had previously informed the local neighborhoods about the visits, questionnaires and blood samplings. Human blood samples were drawn by an official city nurse from owners 18 years of age or older, after they voluntarily provided signed consent. Canine blood samples were obtained by a veterinarian from owned dogs six months of age or older (to avoid biased seropositivity due to maternal antibodies), after obtaining voluntarily signed consent from the owners. Aggressive dogs were not included for blood sampling due to city regulations regarding animal safety.

Sampling analysis was performed by random selection of one person per household. Inclusion criteria included the participation of at least one owner 18 years of age or older and at least one dog older than 6 months of age. Despite cat presence having been recorded on questionnaires for assessment of associated risk of disease, feline serum samplings were not included due to city regulations regarding animal safety.

All blood samples were drawn from July 2015 to July 2016, with both owner and corresponding dog samples and questionnaires taken from the same household on the same day. Serum samples were separated and stored at -20°C until submission for indirect fluorescence IgG anti-*T*. *gondii* antibody testing (IFAT), using tachyzoites of the RH strain as antigen [41] and with a serum dilution of 1:16 (cut-off). Species-specific fluorescein isothiocyanate-labeled conjugates were used (Sigma Chemical Co. and Zimed), as well as positive and negative control sera [42].

Epidemiological analyses were performed based on an epidemiological questionnaire that had been formulated, tested, and applied in previous studies [4,43]. Questionnaires included closed questions on variables associated with human and dog exposure to *T*. *gondii* and were organized into three blocks that queried the main influencing variables for seroprevalence of IgG anti-*T*. *gondii* antibodies: A. socio-economic-environmental variables, B. personal sanitary habits and behavior, and C. animal behavior and management (S1 File).

Variable groups were chosen for three multiple logistic regression models with these dependent variables: seropositivity of household (presence of at least one seropositive owner or dog), owners and dogs. Initially, univariate analysis was performed with estimation of ORs (with confidence intervals of 95%) and chi-squared testing between independent and dependent variables. The selected independent variables and recategorization are presented in S1 Table. Entry of each variable in the multiple logistic regression model had p≤0.20 as a cut-off point, with entry order determined by the p-value and the permanence in the model determined by the significance of estimated coefficients. Ultimately, interactions between independent variables of the final model were tested and include in the final model to evaluate the effect of pairs of independent variables in the final multiple logistic model. These analyses were performed in the R environment.

Maps with point distributions were built using ArcGIS^®^ [44]. Cluster analyses were performed using the scan spatial statistic (SatScan) described by Kulldorff [45], choosing the “Purely Spatial Probability Model” approach, with only high rates. Spatial relative risks were calculated with a significance of 5% [46]. In parallel, a kernel smoothed intensity analysis from a point pattern was performed for positive cases with the density.ppp function of the “spatsat” package in the R environment [47]. The bandwidth was selected based on recent discussion in the literature [48]. This analysis was applied to determine heat areas and for comparison with the clusters. All analyses were made considering three units: human, dog and residence (with at least one seropositive human and/or dog).

Cats were excluded from the present survey due to city regulations regarding employee safety and animal welfare. However, due to the foodborne characteristic of toxoplasmosis, with a strong environmental role for oocyst sporulation, the impact of cat presence in households was statistically investigated as a variable associated with human and dog infection.

## Results

The total number of visits exceeded the minimum sampling calculation of 289/461 (62.67%; 95% CI: 58.19–66.98) households, mainly due to random volunteer requests from neighbors during regular visits. However, failure to obtain biological samples or incomplete questionnaires in 186/289 (64.35%) households led to a final sampling of 564 households.

Overall, a total of 750 households were visited, and sampling surpassed the minimum calculation, 564/461 (122.56%) households, which included 1,985 human beings, 1,170 dogs and 274 cats. From the selected 564 households, a total of 597/1,985 (30.07%; 95% CI: 28.10–32.13) human beings and 729/1,170 (62.30%; 95% CI: 59.49–65.04) dogs were sampled, and spatial distributions were proportionally plotted based on the city population density.

Antibody Testing (IFAT) for IgG anti-*T*. *gondii* antibodies was considered positive in at least one individual (owner or dog) in 244/564 (43.26%; 95% CI: 39.23–47.38) households, with an overall seropositivity of 248/597 (41.54%; 95% CI: 37.65–45.54) owners and 119/729 (16.32%; 95% CI: 13.82–19.18) dogs, with a statistically significant difference between prevalences (OR 3.63; p<0.001). Simultaneous human-dog seropositivity was found in 43/244 (17.62%; 95% CI: 13.35–22.89) households, with only positive human samples in 201/244 (82.37%; 95% CI: 77.11–86.65) households and 64/244 (26.22%; 95% CI: 21.11–32.09) households with only positive dogs.

Multiple analysis showed significant concomitant variables for seropositivity in the household, including public sewer service (p = 0.005), frequency of yard cleaning (p = 0.039) and visualization of accumulated dirt in the yard (p = 0.025). Additionally, having no public sewer service was also associated with an increased risk (95% CI:1.39–6.43), while frequent cleaning of the yard (95% CI:0.49–0.98) and no visualization of dirt in the yard at the time of sampling (95% CI:0.47–0.95) were protective associated factors when associated individually. The interaction among these co-variables was tested, and despite a significant interaction between the frequency of yard cleaning and visualization of accumulated dirt in the yard (OR = 0.48, CI = 0.33–0.69, p-value < 0.0001), when included in the model, no significant variations were produced (Table 1). No significant multiple logistic model was observed for owners, despite a verified association with monthly family income (p = 0.01) by univariate analysis (Table 2).

**Table 1:** Results of univariate logistic regression analysis of 564 households (owners or dogs) IgG anti-*T*. *gondii* antibodies detected by IFAT in the urban area of Londrina from July 2015 to July 2016.

**A: Univariate logistic regression analysis**				
**Household Variables**	**Yes/ total (%)**	**OR**	**95% CI**	**p-value**
*Monthly income (Minimum wage):				
≤ 3 MW	424/564 (75.2)	0.71	0.47–1.06	0.09
> 3 MW	140/564 (24.8)			
Source of drinking water:				
Public system	533/564 (94.5)	0.87	0.39–1.93	0.71
Other	31/564 (5.5)			
Presence of accumulated water at the yard:				
Yes	77/564 (13.7)	1.15	0.69–1.91	0.62
No	487/564 (86.3)			
Water box:				
Yes	493/564 (87.4)	1.06	0.63–1.82	0.89
No	71/564 (12.6)			
Cleaning of water box:				
Presence	124/564 (22.0)	0.99	0.64–1.52	0.98
Abcense	493/564 (65.4)			
*Sewer:				
Public sewer system	524/564 (92.9)	3.02	1.36–7.35	0.01
No public sewer system	40/564 (7.1)			
Lid on water box:				
Yes	483/564 (85.6)	0.83	0.18–3.62	0.76
No	10/564 (1.8)			
Discharge of domestic garbage:				
Plastic bag or garbage can	544/564 (96.5)	1.54	0.56–4.64	0.49
Other	20/564 (3.5)			
Empty lot:				
Yes	300/564 (53.2)	1.05	0.74–1.49	0.79
No	264/564 (46.8)			
*Frequency of yard cleaning:				
Daily	345/564 (61.2)	0.75	0.53–1.07	0.12
Occasionally	219/564 (38.8)			
Presence of cats at the household:				
Yes	457/564 (81.0)	1.16	0.51–1.82	0.51
No	107/564 (19.0)			
*Visualization of accumulated dirt:				
Yes	231/564 (41.0)	0.69	0.48–0.99	0.04
No	333/564 (59.0)			
**B: Final logistic model**				
**Adjusted-OR**	**p-value** **(Wald test)**			
2.99	0.005			
0.69	0.039			
0.67	0.024			

p<0.05, Chi square test, OR: odds ratio, MW: the monthly State Minimum Wage at the time of survey was R$ 880.00, equivalent to U$264.26 with an exchange rate of 3.33 for US$ Dollar to R$ Real.

*variables included in the logistic models.

**Table 2:** Results of univariate logistic regression analysis of 597 owners with IgG anti-*T*. *gondii* antibodies detected by IFAT in the urban area of Londrina from July 2015 to July 2016.

Owners Variables	Yes/ total (%)	OR	(95% CI)	p-value
Gender				
Male	438/597 (73.4)	0.87	0.59–1.29	0.51
Female	158/597 (26.5)			
*Occupation:				
Retired or homework	383/597 (64.2)	0.78	0.54–1.11	0.16
Other	211/597 (35.3)			
*Monthly income:				
< 3 Minimum wage	446/597 (74.7)	0.57	0.38–0.85	0.01
> 3 Minimum wage	151/597 (25.3)			
Hygiene of fruits and vegetables:				
Yes	592/597 (99.2)	0.46	0.01–5.84	0.64
No	4/597 (0.7)			
Washing hands prior to meals:				
Yes	587/597 (98.3)	1.77	0.37–9.00	0.50
No	9/597 (1.5)			
Meat consumption:				
Yes	583/597 (97.7)	0.62	0.14–2.24	0.57
No	13/597 (2.2)			
Raw meat consumption:				
Yes	146/597 (24.5)	0.92	0.62–1.36	0.69
No	450/597(75.4)			
Raw kebab consumption:				
Yes	106/597(17.8)	0.79	0.51–1.24	0.33
No	490/597(82.1)			
Barbecue consumption:				
Yes	196/597(32.8)	1.02	0.71–1.46	0.93
No	400/597(67.0)			
Smoked sausage consumption:				
Yes	472/597(79.1)	1.06	0.69–1.61	0.84
No	124/597 (20.8)			
Fresh sausage consumption:				
Yes	456/597(76.4)	1.11	0.74–1.66	0.62
No	140/597(23.5)			
Salami consumption:				
Yes	328/597(54.9)	1.13	0.80–1.59	0.51
No	268/597(44.9)			
*Soil contact:				
Yes	238/597(39.9)	0.75	0.53–1.06	0.09
No	358/597(60.0)			
Presence of cats:				
Yes	445/597(74.5)	1.23	0.84–1.82	0.29
No	152/597(25.5)			

p<0.05, Chi square test, OR: odds ratio, MW: the monthly State Minimum Wage at the time of survey was R$ 880.00, equivalent to U$264.26 with an exchange rate of 3.33 for US$ Dollar to R$ Real.

* variables included in the logistic models. There was no sufficient N to proceed the analysis.

Multiple analysis in dogs showed that the absence of other dogs (p = 0.001) and the absence of accumulated dirt in the yard (p = 0.028) were significant concomitant variables for dog seropositivity, and both were protective associated factors when analyzed individually (adjusted OR 0.52 and 0.61, respectively). Despite a significant interaction among tested co-variables (OR 1.52, CI 1.10–2.11, p-value = 0.007), no statistical significance was observed when these were included in the multiple model (Table 3).

**Table 3:** Results of univariate logistic regression analysis of 729 dogs with IgG anti-*T*. *gondii* antibodies detected by IFAT in the urban area of Londrina from July 2015 to July 2016.

**A: Univariate logistic regression analysis**				
**Dogs Variables**	**Yes/ total (%)**	**OR**	**(95% IC)**	**p-value**
Monthly income:				
≤ 3 Minimum wage	729/729(100.0)	**		
> 3 Minimum wage	0/729(000)			
Frequency of yard cleaning:				
Daily	448/729(61.5)	1.00	0.65–1.53	0.98
Occasionally	281/729(38.5)			
Presence of cats at the household:				
Yes	141/729(19.3)	0.94	0.56–1.61	0.80
No	588/729(80.7)			
*Presence of other dogs:				
Yes	467/729(64.1)	0.58	0.36–0.91	0.02
No	262/729(35.9)			
*Visualization of accumulated dirt:				
Yes	306/729(42.0)	0.50	0.33–0.76	0.01
No	423/729(58.0)			
Gender:				
Male	407/729(55.8)	0.83	0.54–1.26	0.36
Female	322/729(44.2)			
Reproductive status:				
Neuter / Spayed	103/729(14.1)	1.41	0.75–2.85	0.31
Intact	626/729(85.9)			
Difficulties at birth:				
Yes	589/729(80.8)	0.54	0.18–1.74	0.26
No	24/729(3.3)			
*Raw meat intake:				
Yes	220/729(30.2)	0.72	0.47–1.12	0.13
No	509/729(69.8)			
Age:				
≤ 2 years old	232/729(31.8)	1.20	0.77–1.91	0.45
> 2 years old	497/729(68.2)			
Access to street:				
Yes	387/729(53.1)	1.01	0.66–1.52	0.97
No	342/729(46.9)			
Hunting habit:				
Yes	319/729 (43.8)	1.04	0.69–1.59	0.84
No	410/729(56.2)			
Presence of horses:				
Yes	704/729(96.6)	0.69	0.13–2.35	0.78
No	25/729(3.4)			
Presence of cattle:				
Yes	726/729(99.6)	**		
No	3/729(0.4)			
Presence of opossums:				
Yes	725/729(99.5)	**		
No	4/729(0.5)			
*Presence of birds:				
Yes	685/729(94.0)	2.02	0.92–4.19	0.05
No	44/729(6.0)			
**B: Final logistic model**				
**Dog Variable**	**adjusted-OR**	**95 CI adjusted-OR**	**P-value** **(Wald test)**	
Presence of other dogs	0.52	0.35–0.78	0.001	
Presence of accumulated dirt	0.61	0.39–0.96	0.028	

p<0.05, Chi square test, OR: odds ratio, MW: the monthly State Minimum Wage at the time of survey was R$ 880.00, equivalent to U$264.26 with an exchange rate of 3.33 for US$ Dollar to R$ Real.

*variables included in the logistic models

** there was no sufficient expose and no expose to proceed the analysis.

**Fig 1:**
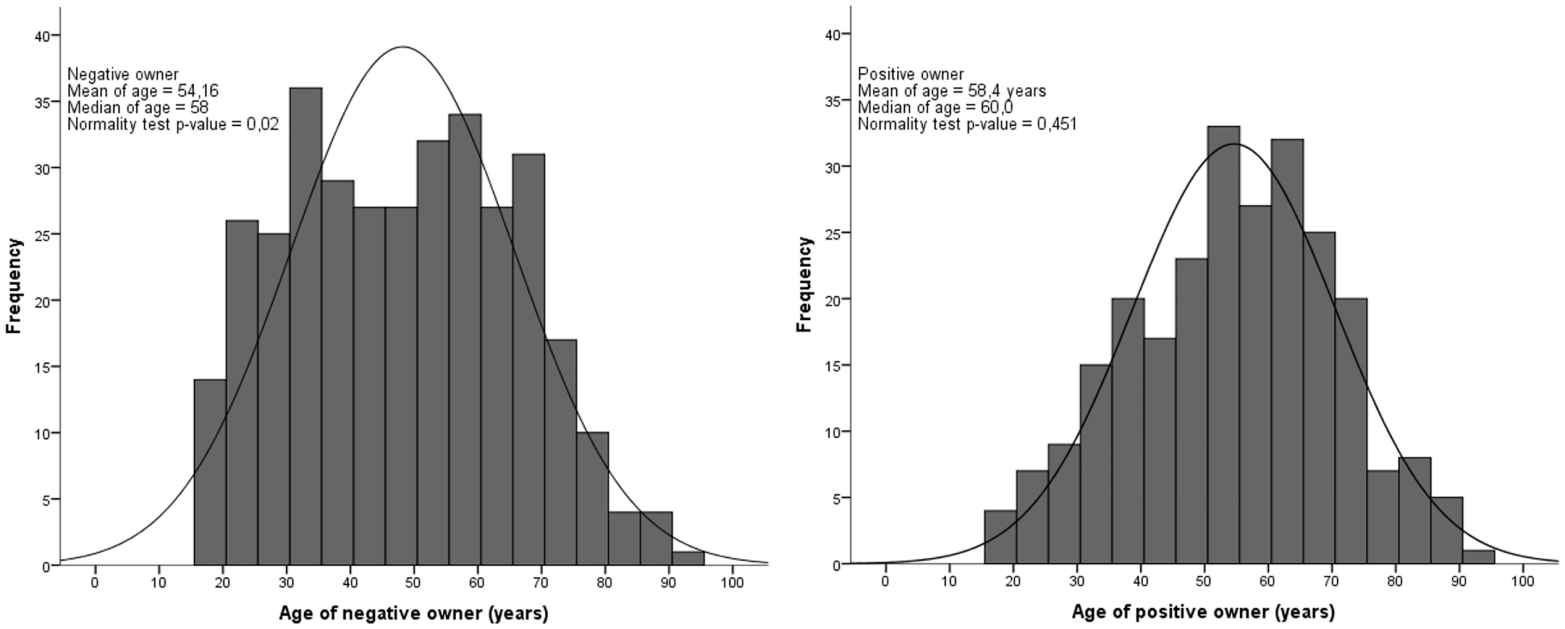
Histogram of age for positive and negative owners for *T*. *gondii* serology in the urban area of Londrina, from July 2015 to July 2016.

**Fig 2:**
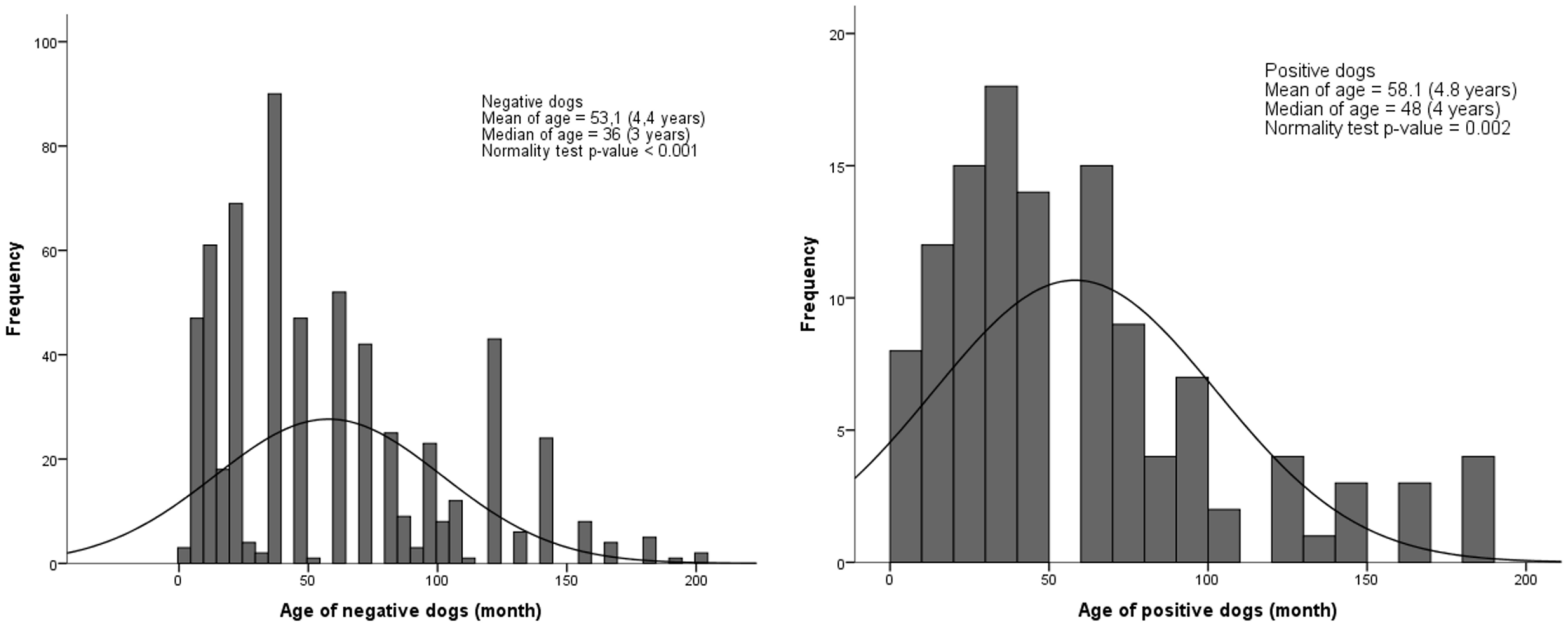
Histogram of age for positive and negative dogs for *T*. *gondii* serology in the urban area of Londrina, from July 2015 to July 2016.

A significant difference was identified for age between seropositive and seronegative owners, with the median age of negative owners being significantly lower than for positive owners (p<0.001) (Fig 1). No significant difference for age was observed between seropositive and seronegative dogs (p = 0.864) (Fig 2).

**Fig 3:**
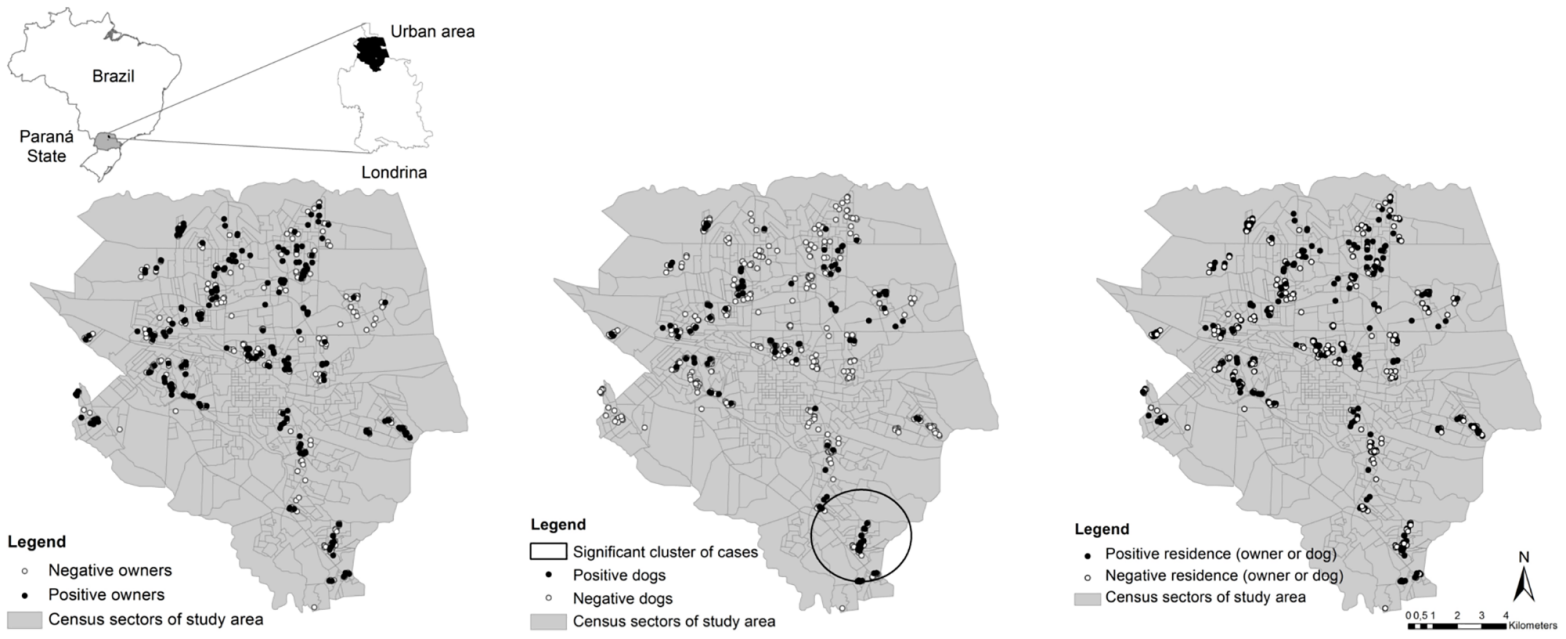
Yesple distribution and relative spatial risk for positive and negative humans, dogs and households (human and/or dogs) for *T*. *gondii* infection in the urban area of Londrina, from July 2015 to July 2016.

**Fig 4:**
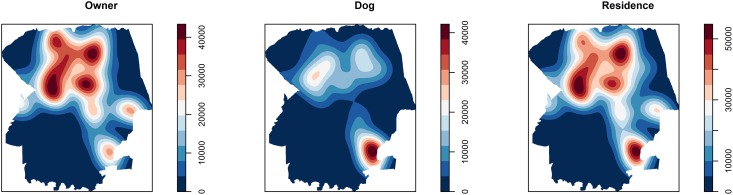
Kernel density analyses of human, dog and household (human and/or dogs) positivity and negativity for *T*. *gondii* infection in the urban area of Londrina, from July 2015 to July 2016.

A significant geographic cluster was found for dogs (RR 3.22; p<0.001) in the southern area (Fig 3), in the same heat area identified by kernel intensity analysis (Fig 4). No significant clusters were found for humans (RR 1.49; p = 0.36) or residences (RR 1.74; p = 0.85), but a heat area was found at the central region. A significant difference (OR 3.52; p<0,001) was detected between the higher prevalence of dogs inside, 32/89 (35.96%; 95% CI: 26.76–46.31), than outside, 88/642 (13.70%; 95% CI: 11.26–16.58), the cluster.

## Discussion

The present study has been, to the authors’ knowledge, the first simultaneous study of *T*. *gondii* seroprevalence in owners and their domiciled dogs. Households where owners and dogs lived were assessed for the potential risk of seropositivity to each other. Although previous studies have shown that infection in companion animals may be accompanied by *T*. *gondii* dissemination in shared habitats with human beings [32,49], a low frequency of households (17.62%) showed simultaneous seropositivity in owners and dogs when compared to solely seropositive owners (82.37%) or dogs (26.22%), suggesting that the intra-domicile environment may have no impact, or at least not a similar impact, between owners and their dogs for *T*. *gondii* infection.

The seroprevalence of human toxoplasmosis, 248/597 (41.54%), measured herein was within previous worldwide reports; however, the prevalence of human toxoplasmosis found in the present study was lower than previous studies in Brazil (S2 Table).

Such comparisons with previous studies should be carefully made, as the results may vary due to serology testing methodology and the cut-offs used [50]. More importantly, previous studies were mostly performed in specific toxoplasmosis risk groups such as pregnant women, children or elderly men, in contrast to the random household approach in the present study [51,52].

Previously reported seroprevalence in dogs has been also focused on specific group populations, such as stray, hunting or owned pet dogs, which vary widely between continents and countries (S2 Table).

Simultaneous intrahousehold analyses of owners and dogs, pioneered in this study, has shown a holistic approach for family health at home, avoiding gaps from separate assessments. Moreover, such spatial methodology should be used for other zoonotic diseases to holistically evaluate the role of the household setting on the disease cycle and epidemiology. Despite previous reports [53–59], the consumption of raw meat and unwashed or raw fruits or vegetables were not associated herein with *T*. *gondii* seropositivity in human beings. Such variation among studies may be explained by climactic, cultural and hygienic disparities of various populations [57].

The foodborne characteristic of toxoplasmosis, which has been primarily transmitted by raw or undercooked meat and contaminated vegetables [2], may be associated with different food exposures of human beings and dogs, which may explain the statistically higher (p<0.01) seroprevalence in owners (41.54%) when compared to their own dogs (16.32%). While owners reported frequent meat consumption (97.70%) and occasional undercooked meat in common local dishes such as barbecue (32.80%), fried meat (24.50%) and raw kibbeh (17.80), 30.20% reported sporadically offering meat to their dogs. In such a scenario with different exposures through food, domiciled dogs may not be considered important sentinels for the infection of their corresponding owners.

Social and economic vulnerability may have played an important role in *Toxoplasma* seroprevalence, since several households reported a higher consumption of raw vegetables and leaves than uncooked meat. Despite previous reports that have already shown that a vegetarian diet may still engender risk for *Toxoplasma* infection [60], further studies should be performed in households within the same area as local markets and restaurants to fully establish the main sources of infection.

The lower seroprevalence in upper income individuals has been consistent with previous studies showing that the associated risk for *T*. *gondii* infection increased 3-fold in low-income and 1.7-fold in medium-income populations but was not an associated risk factor in the upper socioeconomic population [61]. Poor socioeconomic conditions have also been associated with an increased risk for acquiring *T*. *gondii* infection among pregnant women [53,57,62], showing a major geographic distribution impact on parasite transmission in relation to socially and economically deprived areas such as Colombia, South America [63].

In elderly people, the association between low income and *T*. *gondii* seroprevalence has been reported [52], while high socio-economic status was negatively associated [64]. Among low income workers, *T*. *gondii* seropositivity may contribute significantly to workplace accidents. Mental illnesses and behavior alterations, accompanied by cognitive deficits, have been reported as a latent form of toxoplasmosis, which may lead in human patients to workplace accidents [65]. Likewise, as behavior problems have been reported in both human beings and rats [66–68], dogs may also present with such alterations. However, since aggressiveness has not been previously reported in infected dogs, exclusion of aggressive dogs may not have biased the present study. Overall, the presence of a single dog in the household was considered a protective factor associated with *T*. *gondii* infection in dogs. Direct contact with other dogs in the house was found to be associated with infection. Since biting may be a common behavior in dogs, as well as rolling in cat feces [69], the presence of more than one dog increases the associated risk of *T*. *gondii* transmission through direct contact.

Although the presence and density of cats may regulate the intensity of environmental contamination in each area, the presence of infected animals must be accompanied by shed oocysts undergoing sporulation to become infectious [2]. Hence, lack of yard maintenance may lead to dirt and trash accumulation, predisposing to oocyst sporulation and converting intra-domiciliary areas into sources of infection for owners and companion animals.

Geographic characteristics may also influence the dynamics and density of small mammal populations, which have been related via the food chain to dietary responses of several predators including cats [30]. Thus, an absent or insufficient sewer system may directly lead to an increase in the rodent population [70], facilitating hunting and consumption of potentially infected animals by cats.

Although lower than the human seroprevalence (p<0.001), canine *T*. *gondii* antibodies may be due to the consumption of infected vertebrates, as locally detected in 12/46 (26.08%) eared doves by serology [71] and 4/16 (25.00%) rats by PCR [70]. Similar foodborne characteristics may have been the origin of the positive association with trash-filled yards, since failure of yard maintenance may directly provide prolonged *T*. *gondii* persistence or predispose reproduction and colonization of synanthropic animals such as birds and small rodents.

Aging may be associated with the seroprevalence of IgG anti-*T*. *gondii* antibodies due to an increased likelihood of infection over time, in association with lifelong antibody persistence and detection [64]. Not surprisingly, the median human age was significantly higher in seropositive versus seronegative samples for *T*. *gondii* (Fig 1) [72]. Although no statistical differences in age were found between seropositive and seronegative dogs in an urban environment [73–75], comparison among dog populations may be influenced by other variables, such as household characteristics and free street access. As mentioned before, these previous studies were focused on unrelated human or dog seroprevalence and did not consider the relationship between owner-owned dogs and spatial distribution.

Since toxoplasmosis has been reportedly considered a cosmopolitan, foodborne disease, homogeneous distribution of IgG anti–*T*. *gondii* antibodies was expected in owners, as previously shown in pregnant women from Colombia [63] and island dogs of northeastern Brazil [32]. However, when a comprehensive approach was undertaken, the evenly distributed seroprevalence identified may indicate different exposures within city limits, with cluster formation by dogs, as observed via spatial distribution (Figs 3 and 4).

A significant cluster was found in the southern region, which has been urbanized as a consequence of invasion by a landless community currently living with inadequate infrastructure and semi- to non-domiciled dog populations. Such an unsanitary situation may have impacted the significant statistical difference between seropositivity (p<0.001) observed in 32/57 (35.95%) dogs inside this region when compared to 88/642 (13.71%) dogs outside this region, which may be used to estimate the environmental household spread of *T*. *gondii*, as previously reported in free-living cats and dogs [19].

The kernel analysis provided important information, since an opposite dispersion of owners and their dogs was observed (Fig 4), consistent with the limited observation of combined seropositive human and dogs (43.85%), and showed different foodborne characteristics between animal species. Despite being more visual than other analyses, kernel analysis may be limited by not considering the number of samples examined to determine the heat areas.

In the present study, a measurement of population endemicity by region has been created, showing distribution of positive cases throughout the city. Such mapping data may be used as a starting point for further monitoring of toxoplasmosis incidence and prevention, either independently or based on the human:dog ratio.

## Conclusion

In conclusion, characteristics of urban toxoplasmosis may include significantly higher owner seroprevalence than their own dogs, with spatial differences for both human and dog exposures. In addition, no descriptive or spatial evidence was found in this study regarding a potential dog role as sentinels for human toxoplasmosis in urban areas of major cities. Although not a good indicator for human foodborne diseases inside the household, such as toxoplasmosis, dogs may still be a useful sentinel for environmental infection and outbreaks.

Despite toxoplasmosis having been typically considered a foodborne disease, socio-economic factors such as low household income may impact human seroprevalence, along with trash-filled yards with leaves and rubble impacting dog seroprevalence.

## Supporting information

S1 File An instrument used by the Department of Preventive Veterinary Medicine of the Londrina State University, PR to collect epidemiological data, from July 2015 to July 2016, in the city of Londrina, PR. (DOCX)

S1 Table Variables used on multiple analysis in three levels regarding seropositivity for IgG anti-*T*. *gondii* antibodies detected by IFAT in 597 owners and 729 dogs from 564 households (presence of, at least, one positive owner and one positive dog) in the urban area of Londrina from July 2015 to July 2016. (DOCX)

S2 Table Brazilian frequencies of human and canine toxoplasmosis in different profiles and settings. (DOCX)
