# Correction: Spatial and simultaneous representative seroprevalence of anti-*Toxoplasma gondii* antibodies in owners and their domiciled dogs in a major city of southern Brazil

**DOI:** 10.1371/journal.pone.0192570

**Published:** 2018-02-05

**Authors:** Aline do Nascimento Benitez, Felippe Danyel Cardoso Martins, Marcelle Mareze, Nelson Jessé Rodrigues Santos, Fernanda Pinto Ferreira, Camila Marinelli Martins, João Luis Garcia, Regina Mitsuka-Breganó, Roberta Lemos Freire, Alexander Welker Biondo, Italmar Teodorico Navarro

There are two columns missing from Table 1B. There is an error in the Table 1 caption. Please see the corrected Table 1 and caption here.

**Table 1:** Results of univariate and logistic regression analysis of 564 households (owners or dogs) IgG anti-*T*. *gondii* antibodies detected by IFAT in the urban area of Londrina from July 2015 to July 2016.

**A: Univariate analysis**					
**Household Variables**		**Yes/ total (%)**	**OR**	**95% CI**	**p-value**
*	Monthly income (Minimum wage):				
	≤ 3 MW	424/564 (75.2)	0.71	0.47–1.06	0.09
	> 3 MW	140/564 (24.8)			
	Source of drinking water:				
	Public system	533/564 (94.5)	0.87	0.39–1.93	0.71
	Other	31/564 (5.5)			
	Presence of accumulated water at the yard:				
	Yes	77/564 (13.7)	1.15	0.69–1.91	0.62
	No	487/564 (86.3)			
	Water box:				
	Yes	493/564 (87.4)	1.06	0.63–1.82	0.89
	No	71/564 (12.6)			
	Cleaning of water box:				
	Presence	124/564 (22.0)	0.99	0.64–1.52	0.98
	Abcense	493/564 (65.4)			
*	Sewer:				
	Public sewer system	524/564 (92.9)	3.02	1.36–7.35	0.01
	No public sewer system	40/564 (7.1)			
	Lid on water box:				
	Yes	483/564 (85.6)	0.83	0.18–3.62	0.76
	No	10/564 (1.8)			
	Discharge of domestic garbage:				
	Plastic bag or garbage can	544/564 (96.5)	1.54	0.56–4.64	0.49
	Other	20/564 (3.5)			
	Empty lot:				
	Yes	300/564 (53.2)	1.05	0.74–1.49	0.79
	No	264/564 (46.8)			
*	Frequency of yard cleaning:				
	Daily	345/564 (61.2)	0.75	0.53–1.07	0.12
	Occasionally	219/564 (38.8)			
	Presence of cats at the household:				
	Yes	457/564 (81.0)	1.16	0.51–1.82	0.51
	No	107/564 (19.0)			
*	Visualization of accumulated dirt:				
	Yes	231/564 (41.0)	0.69	0.48–0.99	0.04
	No	333/564 (59.0)			
**B: Final logistic regression model**					
		**Adjusted-OR**		**95% CI interval of Adjusted-OR**	**p-value (Wald test)**
Sewer		2.99		1.39–6.43	0.005
Frequency of yard cleaning		0.69		0.49–0.98	0.039
Visualization of accumulated dirt		0.67		0.47–0.95	0.024

p<0.05, Chi square test, OR: odds ratio, MW: the monthly State Minimum Wage at the time of survey was R$ 880.00, equivalent to U$264.26 with an exchange rate of 3.33 for US$ Dollar to R$ Real.

*variables included in the logistic models.

In Table 2, there is information missing from the footnote. There is an error in the Table 2 caption. Please see the corrected Table 2 and caption here.

**Table 2:** Results of univariate and logistic regression analysis of 597 owners with IgG anti-*T*. *gondii* antibodies detected by IFAT in the urban area of Londrina from July 2015 to July 2016.

Owners Variables		Yes/ total (%)	OR	(95% CI)	p-value
	Gender				
	Male	438/597 (73.4)	0.87	0.59–1.29	0.51
	Female	158/597 (26.5)			
*	Occupation:				
	Retired or homework	383/597 (64.2)	0.78	0.54–1.11	0.16
	Other	211/597 (35.3)			
*	Monthly income:				
	< 3 Minimum wage	446/597 (74.7)	0.57	0.38–0.85	0.01
	> 3 Minimum wage	151/597 (25.3)			
	Hygiene of fruits and vegetables:				
	Yes	592/597 (99.2)	0.46	0.01–5.84	0.64
	No	4/597 (0.7)			
	Washing hands prior to meals:				
	Yes	587/597 (98.3)	1.77	0.37–9.00	0.50
	No	9/597 (1.5)			
	Meat consumption:				
	Yes	583/597 (97.7)	0.62	0.14–2.24	0.57
	No	13/597 (2.2)			
	Raw meat consumption:				
	Yes	146/597 (24.5)	0.92	0.62–1.36	0.69
	No	450/597(75.4)			
	Raw kebab consumption:				
	Yes	106/597(17.8)	0.79	0.51–1.24	0.33
	No	490/597(82.1)			
	Barbecue consumption:				
	Yes	196/597(32.8)	1.02	0.71–1.46	0.93
	No	400/597(67.0)			
	Smoked sausage consumption:				
	Yes	472/597(79.1)	1.06	0.69–1.61	0.84
	No	124/597 (20.8)			
	Fresh sausage consumption:				
	Yes	456/597(76.4)	1.11	0.74–1.66	0.62
	No	140/597(23.5)			
	Salami consumption:				
	Yes	328/597(54.9)	1.13	0.80–1.59	0.51
	No	268/597(44.9)			
*	Soil contact:				
	Yes	238/597(39.9)	0.75	0.53–1.06	0.09
	No	358/597(60.0)			
	Presence of cats:				
	Yes	445/597(74.5)	1.23	0.84–1.82	0.29
	No	152/597(25.5)			

p<0.05, Chi square test, OR: odds ratio, MW: the monthly State Minimum Wage at the time of survey was R$ 880.00, equivalent to U$264.26 with an exchange rate of 3.33 for US$ Dollar to R$ Real.

*variables included in the logistic models. There was no significant logistic regression model for this analysis.

In Table 3B, the heading should read: B: Final logistic regression model. There is an error in the Table 3 caption. Please see the corrected Table 3 and caption here.

**Table 3:** Results of univariate and logistic regression analysis of 729 dogs with IgG anti-*T*. *gondii* antibodies detected by IFAT in the urban area of Londrina from July 2015 to July 2016.

**A: Univariate analysis**					
	**Dogs Variables**	**Yes/ total (%)**	**OR**	**95% IC**	**p- value**
	Monthly income:				
	≤ 3 Minimum wage	729/729 (100.0)	**		
	> 3 Minimum wage	0/729 (0.00)			
	Frequency of yard cleaning:				
	Daily	448/729 (61.5)	1.00	0.65–1.53	0.98
	Occasionally	281/729 (38.5)			
	Presence of cats at the household:				
	Yes	141/729 (19.3)	0.94	0.56–1.61	0.80
	No	588/729 (80.7)			
*	Presence of other dogs:				
	Yes	467/729 (64.1)	0.58	0.36–0.91	0.02
	No	262/729 (35.9)			
*	Visualization of accumulated dirt:				
	Yes	306/729 (42.0)	0.50	0.33–0.76	0.01
	No	423/729 (58.0)			
	Gender:				
	Male	407/729 (55.8)	0.83	0.54–1.26	0.36
	Female	322/729 (44.2)			
	Reproductive status:				
	Neuter / Spayed	103/729 (14.1)	1.41	0.75–2.85	0.31
	Intact	626/729 (85.9)			
	Difficulties at birth:				
	Yes	589/729 (80.8)	0.54	0.18–1.74	0.26
	No	24/729 (3.3)			
*	Raw meat intake:				
	Yes	220/729 (30.2)	0.72	0.47–1.12	0.13
	No	509/729 (69.8)			
	Age:				
	≤ 2 years old	232/729 (31.8)	1.20	0.77–1.91	0.45
	> 2 years old	497/729 (68.2)			
	Access to street:				
	Yes	387/729 (53.1)	1.01	0.66–1.52	0.97
	No	342/729 (46.9)			
	Hunting habit:				
	Yes	319/729 (43.8)	1.04	0.69–1.59	0.84
	No	410/729 (56.2)			
	Presence of horses:				
	Yes	704/729 (96.6)	0.69	0.13–2.35	0.78
	No	25/729 (3.4)			
	Presence of cattle:				
	Yes	726/729 (99.6)	**		
	No	3/729 (0.4)			
	Presence of opossums:				
	Yes	725/729 (99.5)	**		
	No	4/729 (0.5)			
*	Presence of birds:				
	Yes	685/729 (94.0)	2.02	0.92–4.19	0.05
	No	44/729 (6.0)			
**B: Final logistic regression model**					
		**adjusted-OR**		**95 CI adjusted-OR**	**p-value (Wald test)**
Presence of other dogs		0.52		0.35–0.78	0.001
Presence of accumulated dirt		0.61		0.39–0.96	0.028

p<0.05, Chi square test, OR: odds ratio, MW: the monthly State Minimum Wage at the time of survey was R$ 880.00, equivalent to U$264.26 with an exchange rate of 3.33 for US$ Dollar to R$ Real.

*variables included in the logistic models.

** there was no sufficient expose and no expose to proceed the analysis.
