# Supplementary material for: Spatial and simultaneous representative seroprevalence of anti-Toxoplasma gondii antibodies in owners and their domiciled dogs in a major city of southern Brazil
Source: PLoS One. 2017 Jul 21;12(7):e0180906. doi: 10.1371/journal.pone.0180906 (PMC5521765; doi:10.1371/journal.pone.0180906)
Supplement: S1 File — (DOCX) [file pone.0180906.s001.docx]

**Supporting information:**

**Supplementary 1 file:** An instrument used by the Department of Preventive Veterinary Medicine of the Londrina State University, PR to collect epidemiological data, from July 2015 to July 2016, in the city of Londrina, PR.

| Id House: | | | | | | | | | | | | | | UBS: | | | | | | | | |
| --- | --- | --- | --- | --- | --- | --- | --- | --- | --- | --- | --- | --- | --- | --- | --- | --- | --- | --- | --- | --- | --- | --- |
| Sector: | Block: | | GPS: | | | | | | | | | | | | | | Date: | | | | | |
| Neighborhood: | | | | | | | | | | | | | | | | | | | | | | |
| Street/Nº: | | | | | | | | | | | | | | | | | | | | | | |
| Name: | | | | | | | | | | | | | | | | | | | | | | |
| Telephone: | | | | | | | | | | | | | | | | | | | | | | |
| **Environmental Questions** | | | | | | | | | | | | | | | | | | | | | | |
| **1.** How much time living in this address? | | | | | | | | | | | **2.** How many people share the house? | | | | | | | | | | | |
| **3.** Family income: (R$ ___________________) (xxx)1. Up to 1 MW (Minimum Wage) | | | | | | | | | | | | | | | | | | | | | | |
|  | | | | (xxx)2. Between 1 to 3 MW | | | | | | | | | (xxx)3. More than 3 MW | | | | | | | | | |
| **4.** Source of Potable water? (xxx) 1. Public (xxx) 2. Well (xxx) 3. Spring water | | | | | | | | | | | | | | | | | | | | | | |
| **5.** When raining, there is water accumulated near home? (xxx) 1.Yes (xxx) 2. No | | | | | | | | | | | | | | | | | | | | | | |
| **6.** There is water reservoir in the house? | | | | (xxx) 1. Yes | | | | | | | | | (xxx) 2. No | | | | | | | | | |
| **7.** The water reservoir is closed? | | | | (xxx) 1. Yes | | | | | | | | | (xxx) 2. No | | | | | | | | | |
| **8.** What frequency the water reservoir is cleaned? | | | | (xxx) 1. One time in 6 months | | | | | | | | | (xxx) 2. One time per year | | | | | | | | | |
|  | | | | (xxx) 3. Don’t clean | | | | | | | | | (xxx) 4. Don’t know the frequency | | | | | | | | | |
| **9.** How is the disposal of wastewater? | | | (xxx) 1. Public | | | | | | | | | (xxx) 2. Cesspit | | | | | | | | (xxx) 3. Don’t have disposal | | |
| **10.** How is the garbage disposal? | | | | | | | | | | (xxx) 1. Plastic bags | | | | | | | | | (xxx) 2.Open Litter | | | |
| (xxx) 3. Plastic bag in litter | | | | | | | | | (xxx) 4. burn | | | | | | | | | | (xxx) 5. Open air | | | |
| **11.** There is unoccupied areas near house? (xxx) 1. Yes (xxx) 2.No | | | | | | | | | | | | | | | | | | | | | | |
| **12.** How many restroom in the house? (xxx)1. One (xxx)2. Two to Four (xxx)3. ≥ Five | | | | | | | | | | | | | | | | | | | | | | |
| **13.** There is restroom outside the house? (xxx) 1. Yes (xxx) 2.No | | | | | | | | | | | | | | | | | | | | | | |
| **14.** which frequency the garden is cleaned? | | | | | | | | | | | | | (xxx) 1. daily | | | | | | | | | (xxx) 2. weekly |
|  | | | | | | | | (xxx) 3. biweekly | | | | | (xxx) 4. Monthly | | | | | | | | (xxx) 5. Don’t do | |
| **15.** The garden has: | | (xxx)1. Garbage | | | (xxx)2.Tree leaves | | | | | | (xxx) 3. Construction debris ( )4. cleaned | | | | | | | | | | | |
| **16**. Have you ever seen rats in the house? | | | | (xxx) 1. Yes, inside de house | | | | | | | | | (xxx) 2. Yes, in the garden (xxx)3. No | | | | | | | | | |
| **17.** How many rats together? | | | | (xxx) 1.One (xxx) 2. Two (xxx)3. Three (xxx) 4. Four (xxx) 5. ≥ Five | | | | | | | | | | | | | | | | | | |
| **18.** What do you use to control ratss? (xxx)1. Cats | | | | | | | | | | | (xxx)2. Poison (xxx)3. mousetrap (xxx)4. Nothing | | | | | | | | | | | |
| **19.** Have you ever seen ticks in the house? (xxx)1. Yes, in the backyard (xxx)2. Yes, inside the house (xxx)3. No | | | | | | | | | | | | | | | | | | | | | | |
| **20.** In which season does it appear? | | | | | | | | | | | | |  | | | | | | | | | |
|  | | | | | | (xxx)1. 01/dec to 28/feb (Summer) | | | | | | | | | (xxx)3. 01/jun to 31/aug (Winter) | | | | | | | |
|  | | | | | | (xxx)2. 01/mar to 31/may (Autumn) | | | | | | | | | (xxx)4. 01/set to 30/nov (Spring) | | | | | | | |
| **21.** Has Mosquitoes in the house? (xxx)1. Few (xxx)2. A lot (xxx) Don’t | | | | | | | | | | | | | | | | | | | | | | |
| **22.** How do you control mosquitoes? | | | | | | | | (xxx)1. Net in the window | | | | | | | | | | (xxx)2. Repellent in the body | | | | |
|  | | | | | | | | (xxx)3. Repellent in the environment | | | | | | | | (xxx)4. Don’t control | | | | | | |
| **23.** How many dogs and cats are in the house? | | | | | | | Dogs: Cats: | | | | | | | | | | | | | | | |
| Describe below which animals will participated in the study: | | | | | | | | | | | | | | | | | | | | | | |
| \| An. \| Esp. \| Name \| Animal ID \| An. \| Esp. \| Name \| Animal ID \| \| --- \| --- \| --- \| --- \| --- \| --- \| --- \| --- \| \| **1** \|  \| **xxxxxxxxxxxxxxxx** \| **Xxxxxxxxxxxxxx** \| **5** \|  \| **xxxxxxxxxxxxxxxx** \| **xxxxxxxxxxxxxx** \| \| **2** \|  \|  \|  \| **6** \|  \|  \|  \| \| **3** \|  \|  \|  \| **7** \|  \|  \|  \| \| **4** \|  \|  \|  \| **8** \|  \|  \|  \| | | | | | | | | | | | | | | | | | | | | | | |

| **QUESTÕES SÓCIO ECONÔMICAS** | | | | | | | | | UBS: | | | | | | | | | |
| --- | --- | --- | --- | --- | --- | --- | --- | --- | --- | --- | --- | --- | --- | --- | --- | --- | --- | --- |
| House number | | Person number: | | | | | | | | | | | | Date: | | | | |
| Name of volunteer: | | | | | | | | | | | | | | | | | | |
| Gender: (xx)1. F (xx)2. M | Age: | | | | | | | Occupation: | | | | | | | | | | |
| **24.** How long are you living in this house? | | | | |  | | | | | | | | | | |  | | |
| (xxx)1. up to five years | | | (xxx)2. from five to ten years | | | | | | | | | | | | | (xxx)3. More than tem years | | |
| **25.** Do you clean fruits and vegetables before eat? | | | | | | | | | | | | | | | |  | | |
| (xxx)1. Yes, always | | | (xxx)2. Yes, almost always | | | | | | | | | | | | | (xxx)3. No | | |
| **26.** What do you use to clean? | | | | | | | | | | | | | | | |  | | |
| (xxx)1. Only water | | | (xxx) 2. Bleach | | | | | | | | | | | | | (xxx) 3. Vinegar | | |
| **27.** Do you wash your hands before eat? | | | | | | | | | | | | | | | |  | | |
| (xxx)1. Always, with water and soap  (xxx)2. Almost always, with water and soap | | | | | | | (xxx)3.Almost always, water  (xxx)4. Sometimes | | | | | | | | | | (xxx) 5. No | |
| **28.** Do you wash your hands after go to the restroom? | | | | | | | | | | | | | | | | | | |
| (xxx)1. Always, with water and soap  (xxx)2. Almost always, with water and soap | | | | | | | (xxx)3.Almost always, water  (xxx)4. Sometimes | | | | | | | | | | (xxx) 5. No | |
| **29.** What kind of meat do you eat? (xxx)1. Beef | | | | | | | | | | | (xxx)2. Pork | | | | | (xxx) 3. Lamb | | |
|  | | | (xxx) 4. Chicken | | | | | | | | (xxx)5. Fish | | | | | (xxx)6. Don’t eat meat | | |
| **30.** Do you eat meat raw or rear? | | | | | | (xxx)1. Yes | | | | | (xxx)2. No | | | | |  | | |
| Do you have the habit to eat: | | | | | | | | | | | | | | | |  | | |
| **31.** Raw Kebab | | | (xxx)1. Yes | | | | | | | | | | | | | (xxx)2. No | | |
| **32.** Rear Barbecue | | | (xxx)1. Yes | | | | | | | | | | | | | (xxx)2. No | | |
| **33.** Smoked Sausage | | | (xxx)1. Yes | | | | | | | | | | | | | (xxx)2. No | | |
| **34.** Fresh sausage | | | (xxx)1. Yes | | | | | | | | | | | | | (xxx)2. No | | |
| **35.** Homemade salami | | | (xxx)1. Yes | | | | | | | | | | | | | (xxx)2. No | | |
| **36.** Raw Milk? | | | (xxx)1. Yes | | | | | | | | | | | | | (xxx)2. No | | |
| **37.** Do you have frequent contact with soil or sand? | | | | | | | | | | | | | (xxx)1. Yes | | | | | (xxx)2. No |
| **38.** Do you visit forest areas? | | | (xxx)1. Yes, every day | | | | | | | | | | | | | (xxx)2. Yes, one time per week | | |
| (xxx)3. Yes, one time per month | | | (xxx)4. Yes, one time per year | | | | | | | | | | | | | (xxx)5. No | | |
| **39.** Have you ever see ticks in your body? | | | | | | | | | | | | (xxx)1. Yes | | | | (xxx)2. No | | |
| **40.** Were you hospitalized last year because of illness?? | | | | | | | | | | | | (xxx)1. Yes | | | | (xxx)2. No | | |
| **41.** Do you know what disease was? (xxx)1. Yes (xxx)2. No Qual? | | | | | | | | | | | | | | | | | | |
| **42.** Do you have diarrhea during last week? | | | | | | | | | | | | (xxx)1. Yes | | | | (xxx)2. No | | |
| **43.**  Have you ever had hepatitis or jaundice? (xxx)1. Yes (xxx)2. No (xxx)3. Don’t know | | | | | | | | | | | | | | | | | | |
| **44.** Do you have any lesion in skin? | | | | | | (xxx)1. Yes (xxx)2. No | | | | | | | | | Local: | | | |
| **45.** How many time do you have the lesion? | | | |  | | | | | | | | | | |  | | | |
| **46.** What kind in this lesion? | | | | (xxx)1. Nodular (xxx)2. Ulcerada | | | | | | | | | | |  | | | |
| **47.** Have you traveled in last 12 months? | | (xxx)1. Yes, | | | | | | | | (xxx)2. No | | | | | | | | |
| **48.** Which destination? | | | |  | | | | | | | | | | |  | | | |

| **Questions about animal** | | | | | | | | | | | UBS: | | | | | | | | | | |
| --- | --- | --- | --- | --- | --- | --- | --- | --- | --- | --- | --- | --- | --- | --- | --- | --- | --- | --- | --- | --- | --- |
| House number: | | | | | Animal ID | | | | | | | | | | | | Date: | | | | |
| Owner name: | | | | | | | | | | | | | | | | | | | | | |
| Animal name: | | | | | | Species: (xxx)Can (xx)Fel | | | | | | | | | | Gender:(x)1.F (x)2.M | | | | Age: | |
| **49.** Is it castrated? (xxx)1. Yes (xxx)2. No | | | | | | | | | **50.** Already bred? (xxx)1. Yes (xxx)2. No | | | | | | | | | | | | |
| **51.** What is the origin of the animal? | | | | | | | | | | | | | | | | | | | | | |
| **52.** Does it have any parturient disorder? (xxx)1. Yes (xxx)2. No | | | | | | | | | | | | | | Which one? | | | |  | | | |
| **53.** Is it feed with raw or rear meat? | | | | | | | | | | | | | | (xxx)1. Yes | | | (xxx)2. No | | | | |
| **54.** Has the vaccination card control? | | | | (xxx)1. Yes | | | | (xxx)2. No | | | | | | | | | | | | | |
| **55.** When the animal received the last vaccine? | | | | | | | (xxx)1. less than 6 months | | | | | | | | | | (xxx) 2. 6 to 12 months | | | | |
| (xxx) 3. More than12 months | | | | | | | (xxx) No vaccine | | | | | | | | | | (xxx) Don’t remember | | | | |
| **56.** Does the animal has contact with other animals? | | | | | | | (xxx)1. Yes, dogs | | | | | | | | | | (xxx)2. Yes, cats | | | | |
| (xxx)3. Yes, equids | (xxx)4. Yes, bovines | | | | | | (xxx)5. Yes, raccon | | | | | | | | | | (xxx)6. Yes, birds | | | | (xxx) 7. No |
| **57.** Does the animal has access to the street? | | (xxx)1. Yes, free | | | | | | | | (xxx)2. Yes, partly | | | | | | | | | (xxx)3. No | | |
| **58.** Does the animal has hunt habits? | | (xxx)1. Yes | | | | | (xxx)2. No | | | | | | | | | | | | | | |
| **59.** Has had vomiting / diarrhea in the past 30 days? | | | | | | | | | | (xxx)1.Yes, vomiting | | | | | | | (xxx)2. Yes, diarrhea | | | | |
|  | | | | | | | | | | (xxx)3. Yes, both | | | | | | | (xxx)4. No | | | | |
| **60.** Has lose weight in last 30 days? | | | | | | | (xxx)1. Yes | | | | | | (xxx)2. No | | | | | | | | |
| **61.** Has any lesion in the skin? Where? | | | | | | | (xxx)1. Ear | | | | | | | | | | (xxx)2. Snout | | | | |
|  | (xxx)3. Abdomen | | | | | | (xxx)4. scrotum | | | | | | | | | | (xxx)5. Perivulvar/ penis | | | | |
| (xxx)6. Perianal | (xxx)7.Periocular | | | | | | (xxx)8. limbs | | | | | | | | | | (xxx) 9. No | | | | |
| **62.** How is this lesion? | | | (xxx)1. Ulcerate (xxx)2. Nodular | | | | | | | | | | | | | | (xxx)3. Diffuse | | | | |
| **63.** How many time has this lesion? | | | | | (xxx)1. < 1 week | | | | | | | | | (xxx)2. 1 to 4 weeks (xxx)3. 1 to 6 months | | | | | | | |
|  | | | | | (xxx)4. 6 to 12 months | | | | | | | | | (xxx)5. More than a year | | | | | | | |
| **64.** Uses repellent collar? | | | (xxx)1. Yes | | | | | | | | | | | | | | (xxx)2. No | | | | |
| **65.** Does it visit forest areas? | | | (xxx)1. Yes, always ( )2. Sometimes | | | | | | | | | | | | | | (xxx)3. No | | | | |
| **66.** Does it has thick? | | | (xxx)1. Yes | | | | | | | | | | | | | | (xxx)2. No | | | | |
| **67.** In which season they appear? (xxx)1. 01/dez to 28/fev (Summer) | | | | | | | | | | | | | | | | | (xxx)3. 01/jun to 31/ago (winter) | | | | |
| (xxx)2. 01/mar to 31/mai (Autunm) (xxx)4. 01/set to 30/nov (spring) | | | | | | | | | | | | | | | | | | | | | |
| **68.** How do you control thicks in the animals? | | | | | | | | | | | | (xxx) 1. Pour on | | | | | (xxx) 2. whash | | | | |
|  | | | (xxx)3. take off by hand | | | | | | | | | (xxx) 4. Don’t control | | | | | (xxx) 5. another | | | | |
| **69.** What frequency? | | | | | (xxx)1. monthly | | | | | | | | | | (xxx) 2. Biyearly | | | | | | |
| (xxx)3. yearly | | | | | (xxx)4. Sporadically | | | | | | | | | | (xxx) 5. Don’t’do | | | | | | |
| **70.** Has fleas? | | | | | (xxx)1. Yes | | | | | (xxx)2. No | | | | | | | | | | | |
| **71.** Does it has locomotion disability? | | | | | (xxx)1. Yes | | | | | (xxx)2. No | | | | | | | | | | | |
