# Supplementary material for: Spatial and simultaneous representative seroprevalence of anti-Toxoplasma gondii antibodies in owners and their domiciled dogs in a major city of southern Brazil
Source: PLoS One. 2017 Jul 21;12(7):e0180906. doi: 10.1371/journal.pone.0180906 (PMC5521765; doi:10.1371/journal.pone.0180906)
Supplement: S1 Table — (DOCX) [file pone.0180906.s002.docx]

**Supporting information:**

**Supplementary 1 table:** Variables used on multiple analysis in three levels regarding seropositivity for IgG anti-*T. gondii* antibodies detected by IFAT in 597 owners and 729 dogs from 564 households (presence of, at least, one positive owner and one positive dog) in the urban area of Londrina from July 2015 to July 2016.

| **Variable** | **Categories** | **Variable** | **Categories** |
| --- | --- | --- | --- |
| **Household** |  | **Dogs** |  |
| Monthly income  (Minimum wage) | 1. Up to 3 MW  2. Above 3MW | Owner monthly income (Minimum wage) | 1. Up to 3 MW  2. Above 3MW |
| Source of drinking water | 1. Public  2. Other sources | Frequency of Yard cleaning | 1. Daily  2. Occasionally |
| Presence of accumulated water on yard | 1. Yes  2. No | Presence of cats at the household | 1. Yes  2. No |
| Cleaning of water box | 1. Yes  2. No | Presence of other dogs besides tested | 1. Yes  2. No |
| Sewer | 1. Public sewer system  2. No public sewer system | Visualization of accumulated dirt | 1. Yes  2. No |
| Discharge of domestic garbage | 1. Plastic bag or garbage can  2. Other | Gender | 1. Male  2. Female |
| Empty lot | 1. Yes  2. No | Reproductive status | 1. Neuter / Spayed  2. Intact |
| Frequency of yard cleaning | 1. Daily  2. Occasionally | Difficulties at birth | 1. Yes  2. No |
| Presence of cats at the household | 1. Yes  2. No | Raw meat intake | 1. Yes  2. No |
| Visualization of accumulated dirt | 1. Yes  2. No | Age | 1. ≤ 2 years old  2. > 2 years old |
| **Owners** |  | Access to street | 1. Yes  2. No |
| Gender | 1. Male  2. Female | Hunting habit | 1. Yes  2. No |
| Occupation | 1. Retired or homework  2. Other | Presence of horses | 1. Yes  2. No |
| Hygiene of fruits and vegetables | 1. Yes  2. No | Presence of cattle | 1. Yes  2. No |
| Hand washing prior to meals | 1. Yes  2. No | Presence of opossums | 1. Yes  2. No |
| Meat consumption | 1. Yes  2. No | Presence de birds | 1. Yes  2. No |
| Raw meat consumption | 1. Yes  2. No |  |  |
| Raw kebab consumption | 1. Yes  2. No |  |  |
| Barbecue consumption | 1. Yes  2. No |  |  |
| Smoked sausage consumption | 1. Yes  2. No |  |  |
| Fresh sausage consumption | 1. Yes  2. No |  |  |
| Salami consumption | 1. Yes  2. No |  |  |
| Contact with soil | 1. Yes  2. No |  |  |
| Presence of cats | 1. Yes  2. No |  |  |
