# Supplementary material for: Spatial and simultaneous representative seroprevalence of anti-Toxoplasma gondii antibodies in owners and their domiciled dogs in a major city of southern Brazil
Source: PLoS One. 2017 Jul 21;12(7):e0180906. doi: 10.1371/journal.pone.0180906 (PMC5521765; doi:10.1371/journal.pone.0180906)
Supplement: S2 Table — (DOCX) [file pone.0180906.s003.docx]

**Supporting information:**

**Supplementary 2 table:** Brazilian frequencies of human and canine toxoplasmosis in different profiles and settings.

| **HUMANS** | | | | | **DOGS** | | | | |
| --- | --- | --- | --- | --- | --- | --- | --- | --- | --- |
| **Country/City**  **(Reference)** | **Sample Year** | **Target Population** | **Seroprevalence**  **Pos/Tot. (%)** | | **Country/City**  **(Reference)** | **Sample Year** | **Target Population** | **Seroprevalence**  **Pos/Tot. (%)** | |
|  |  |  |  |  |  |  |  |  |  |
| Brazil (34) | 2002 | Children | 110/339 | (32.44) | China (41) | 1998-15 | Domiciliated | 5,187/78,719 | (6.58) |
| Brazil (37) | 2005 | Pregnant women | 3345/4883 | (68.50) | Brazil/Southeastern (46) | 2004 | Domiciliated | 116/670 | (17.31) |
| Brazil (35) | 2006 | Pregnant women | 242/492 | (49.18) | Brazil/Southern (47) | 2004-12 | Birth Control Project | 56/271 | (20.66) |
| German (32) | 2008-11 | Adults | 3602/6564 | (55.87) | Mexico | 2006 | Shelter | 52/101 | (51.48) |
| Brazil (36) | 2010-11 | Parturient women | 234/400 | (58.50) | Brazil/Southern and Southeastern (48) | 2006-07 | Domiciliated | 89/400 | (22.25) |
| Italy (31) | 2010-13 | Clinical Over 1 Year | 3476/12306 | (28.20) | Brazil/Northern (49) | 2009-10 | Rural and urban | 55/105 | (52.38) |
| Canada (30) | 2011 | Over 4 Years | 28/201 | (13.90) | Canada (30) | 2010-11 | Clinic | 10/47 | (21.27) |
| Brazil (39) | 2011-12 | Elderly Men | 527/599 | (87.97) | Brazil/Northern and Northeastern (45) | 2011-12 | Domiciliated | 55/476 | (11.55) |
| Brazil (38) | 2012 | Pregnant women | 661/963 | (68.63) | Italy (42) | 2013-14 | Hunting | 94/398 | (23.61) |
| Mexico (28) | 2013-14 | Occupationally Exposed | 12/200 | (6.00) | Spain (43) | 2013-15 | Clinic | 203/664 | (30.57) |
| Indonesia (33) | 2014 | 15-60 Years | 394/630 | (62.54) | Morocco (43) | 2013-15 | Clinic | 5/19 | (26.31) |
| Iran (29) | 2015 | Women | 31/300 | (10.33) |  |  |  |  |  |
